# Hospital Saturday Fund

**Published:** 1903-05-02

**Authors:** 


### HOSPITAL SATURDAY FUND.

The annual meeting of this Fund was held at the Mansion House on April 25th.

The Lord Mayor, who presided, said he was very glad to note that the year 1902 showed an improvement in every respect over 1901, and if this were maintained during the present year, the managers would have matter to congratulate themselves upon. The Hospital Saturday Fund differed in some particulars from the Hospital Sunday Fund and King Edward's Fund, for although all three funds subsidised charitable institutions, they received support from totally different sources. The Saturday Fund was looked upon with increasing favour by the working classes. In addition to the actual amount of money collected, this Fund did a great deal of indirect good, in alliance with the other two funds, by making careful investigations into the management of every institution before sanctioning the contribution of funds. The public attached more and more weight to the certificate given by this Fund when granting contributions, which were not allocated to institutions which did not deserve them by reason of their bad management. His lordship then dwelt upon some of the chief points in the report which showed that the year 1902 was a record year, there being an increase in income of £1,417, which made the total exceed that of any previous year. The amount distributed was also higher than ever before. The distribution committee had made during the year special arrangements for the treatment of persons suffering from consumption. The surgical appliances committee supplied 3,689 instruments, the patients, who greatly appreciated this department of the work, contributing £1,982 towards the cost. Good work had also been done by the ambulance committee. Notwithstanding the large increase in the work of the Fund, the percentage of the expenses of management had decreased.

Sir Savile Crossley, M.P., Chairman of the Fund, who was the next speaker, in recalling the allusion made by the Lord Mayor to the differences of the three funds, said that they agreed in one characteristic, and that was in believing it to be far better to distribute funds to the various institutions as the need arose rather than to allot a certain sum to each hospital for the rest of its existence. S[illegible]vile Crossley went on to say that he looked upon his re-election as Chairman as a great privilege, and considered there was one advantage to be gained by his filling that post, which was that he was also one of the honorary secretaries of the Sunday Fund and on the Distribution Committee of the King's Fund. It was most important that these three funds should work together as much as possible, and the best means of ensuring this was by having identical members on all three boards of management. The Committee felt great satisfaction that the abolition of street collection, so far from causing a loss to the Fund, had had, it was believed, a good effect upon the income. The collections for the Fund being almost entirely made up of small contributions from the working classes, two great benefits were thus conferred by the Fund: persons of limited means were enabled to contribute to its support and habits of thrift were encouraged among the poorer classes. Patients willingly contributed towards the cost of the surgical appliances, and where these were costly payment was made by instalments.

Alderman Sir William Treloar proposed a resolution approving of the efforts of the Hospital Saturday Fund to interest the working classes in the work of the Medical Charities of London, and also its efforts to circulate reliable information as to the prevention and cure of consumption and other diseases. The Alderman in a brief speech advanced the opinion that the maintenance of hospitals ought to be kept up out of the rates.

Sir Edmund Hay Currie, who seconded this resolution, spoke in terms of high admiration of the report, which he considered a marvellous compendium of good work done from one end of London to another. Sir Edmund Currie was not in agreement with the last speaker that it would be desirable for the hospitals to come upon the rates, but said that the time was approaching when that state of things would most probably come about unless the hospitals would make up their minds to keep down expenses. Looking back over the last ten years the increase in the cost of each patient was most startling, and however great the efforts to raise money might be they would find the expenses would go up correspondingly. There was, however, a wonderful difference in the cost of patients at various hospitals. The time had come for the committees of the three funds to put their foot down and refuse to countenance expenditure that exceeded a certain limit. A model hospital in the way of economy was the French Hospital. With regard to consumption, of which mention was made in the resolution, Sir Edmund Currie spoke very strongly on the difficulty that existed in getting a consumptive patient into a hospital, the length of time that elapsed before this could be done, being from six weeks to three months. Ten per cent. of the deaths in London were caused by consumption, which outnumbered those by small-pox and fevers put together. It was a curable disease, but through there being no place to send patients to they frequently did not begin to undergo treatment till their condition was hopeless. Sir Edmund Currie expressed himself strongly in favour of open-air treatment. There was one body, he continued, who might provide the much-needed accommodation, and that was the Metropolitan Asylums Board, who had made large provision for small-pox patients, which was now no longer needed.

The Lady Mayoress then distributed diplomas of merit and first-aid and nursing certificates to the successful students, and a vote of thanks having been passed to the Lord Mayor and Lady Mayoress, the Lord Mayor, in responding, referred to the suggestion that had been made as to the hospitals being kept up by the rates, and said that he should consider that a very deplorable thing, but he thought a medium course might be taken, and at all events those hospitals which were [illegible]perly conducted and sanctioned by the managers of these funds should be exempted from paying rates. His lordship then cited an instance of a hospital which contributed £1,250 to the rates per annum, this sum making the difference between the hospital being self-supporting or dependent on outside contributions.

At the close of the meeting, a lecture, illustrated by magic-lantern slides, was delivered by Dr. T. D. Lister, on "Infant Feeding and Milk Supply."

